# In silico analysis of enantioselective binding of immunomodulatory imide drugs to cereblon

**DOI:** 10.1186/s40064-016-2761-9

**Published:** 2016-07-19

**Authors:** Takahiro Murai, Norihito Kawashita, Yu-Shi Tian, Tatsuya Takagi

**Affiliations:** Graduate School of Pharmaceutical Sciences, Osaka University, 1-6 Yamadaoka, Suita, Osaka 565-0871 Japan; Research Institute for Microbial Diseases, Osaka University, 3-1 Yamadaoka, Suita, Osaka 565-0871 Japan; Graduate School of Information Science and Technology, Osaka University, 1-5 Yamadaoka, Suita, Osaka 565-0871 Japan

**Keywords:** IMiDs, Docking simulation, Cereblon, Enantiomeric selectivity, Teratogenicity

## Abstract

**Background:**

Thalidomide and its analogs, lenalidomide and pomalidomide (referred to as immunomodulatory imide drugs or IMiDs) have been known to treat multiple myeloma and other hematologic malignancies as well as to cause teratogenicity. Recently the protein cereblon was identified as the primary target of IMiDs, and crystallographic studies of the cereblon–IMiDs complex showed strong enantioselective binding for the (*S*)-enantiomer of IMiDs.

**Results:**

Using the structures of cereblon and IMiDs [both (*S*)-enantiomers and (*R*)-enantiomers] we performed docking simulations in order to replicate this enantiomeric selectivity and to identify the region(s) contributing to this selectivity. We confirmed the enantioselective binding of IMiDs to cereblon with high accuracy, and propose that the hairpin connecting the β10–β11 region of cereblon (residues 351–355) contributes to this selectivity and to the increased affinity with IMiDs.

**Conclusions:**

Our docking results provide novel insights into the binding mode of IMiD-like molecules and contribute to a deeper understanding of cereblon-related biology.

**Electronic supplementary material:**

The online version of this article (doi:10.1186/s40064-016-2761-9) contains supplementary material, which is available to authorized users.

## Background

**Fig. 1:**
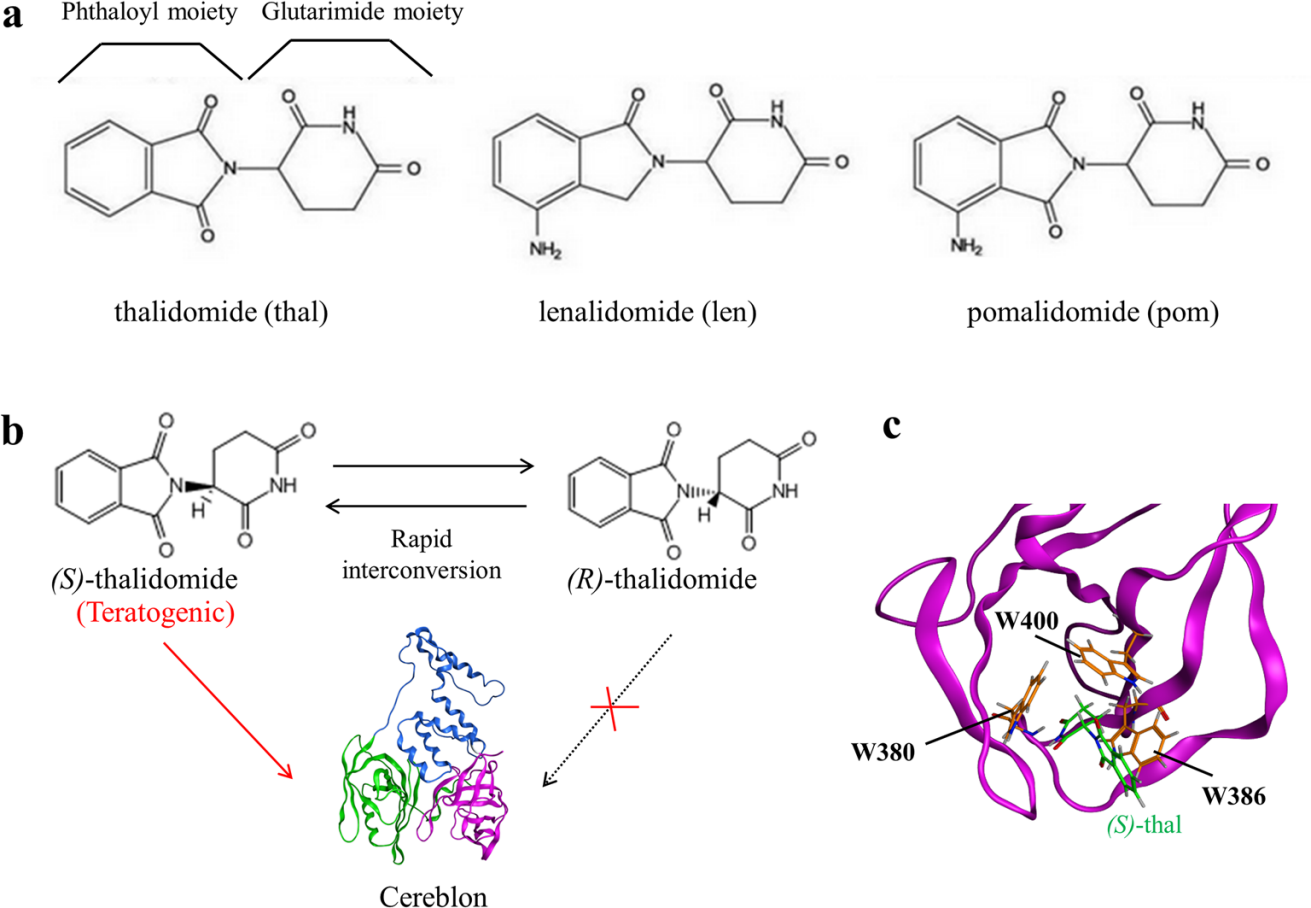
Structures of IMiDs and cereblon. **a** Structures of thalidomide, lenalidomide, and pomalidomide. **b** Schematic representation of enantiomeric selectivity for IMiDs. The NTD, HBD, and CBD domains of cereblon are shown in *green*, *blue*, and *magenta*, respectively. **c** Binding mode of IMiDs to cereblon. The CBD domain of the protein is shown in *magenta*, and the three tryptophans of the aromatic cage (W380, W386, and W400) and (*S*)-enantiomer of thalidomide [(*S*)-thalidomide] are shown in *orange* and *green*, respectively (PDB ID: 4CI1). Thalidomide, lenalidomide, and pomalidomide are described in ‘thal’, ‘len’, ‘pom’ for short, respectively

In the 1950s, thalidomide [α-(*N*-phthalimido)glutarimide] was introduced and taken by many pregnant women as a sedative/anti-nausea drug (Bartlett et al. 2004; Ito et al. 2011; Shortt et al. 2013). In the early 1960s, however, the drug was banned from the market because of its teratogenic potential (Mcbride 1961; Lenz et al. 1962). Despite this notorious effect, intensive research has been carried out with thalidomide due to its efficacy of inhibiting tumor necrosis factor (TNF)-α secretion and treating multiple myeloma and other hematologic malignancies (Sheskin 1965; Singhal et al. 1999). In this context, attempts to augment the effect of the drug resulted in the development of its analogs, lenalidomide and pomalidomide. This class of compounds is referred to as immunomodulatory imide drugs or IMiDs, and these compounds share two structural elements, the glutarimide moiety and the phthaloyl moiety (Fig. 1a). Although apremilast was approved as an analog of IMiDs in 2014 by FDA, apremilast was not included in the current study. Because while both apremilast and thalidomide share a phthaloyl moiety structure, apremilast lacks the glutarimide moiety and thus fails to bind to cereblon, the target of thalidomide action.

Due to chirality, the drugs have two isomeric forms and it is difficult to isolate one enantiomer from the other, because both enantiomers rapidly interchange in vivo (Eriksson et al. 1995; Lepper et al. 2006). Since the discovery of the teratogenic potential of IMiDs, a number of studies have proposed many hypotheses about their mechanisms, including oxidative stress (Parman et al. 1999) and anti-angiogenesis (Therapontos et al. 2009). These efforts, however, have not completely elucidated the mechanism of IMiDs-induced teratogenicity and other therapeutic mechanisms (Bartlett et al. 2004; Ito et al. 2011). In 2010, the discovery that the protein cereblon was the primary target of IMiDs opened a new avenue in IMiD research (Ito et al.). Cereblon protein has three domains; the amino terminal domain (NTD), the α-helical bundle domain (HBD), and the carboxy-terminal domain (CBD), and its sequence is highly conserved between different species (Ito et al. 2011; Shortt et al. 2013). This protein is a part of damage-specific DNA binding protein 1 (DDB1)/cullin4 E3 ubiquitin ligase complex and acts as the recruitment site for the ubiquitylation of substrate proteins, thus promoting their degradation (Ito et al. 2011; Shortt et al. 2013). Recently, the crystal structures of cereblon bound to IMiDs were solved and these structures revealed that the (*S*)-enantiomers of IMiDs bind to the protein, and the myeloid ecotropoic viral insertion site homeobox 2 (MEIS2) protein was identified as a substrate of cereblon (Fischer et al. 2014; Chamberlain et al. 2014) (Fig. 1b). A detailed structural analysis revealed that the glutarimide moiety of these compounds recognizes three tryptophans (W380, W386, and W400) of the aromatic cage (tri-Trp pocket), which are a part of the Tbk1/Ikki binding domain (TBD), and the phthalimide moiety is exposed to the solvent (Fischer et al. 2014; Chamberlain et al. 2014) (Fig. 1c).

Similar to cereblon protein, a number of proteins contain such aromatic cages, suggesting that this protein is bound by other ligands (particularly endogenous ligands) (Chamberlain et al. 2014; Hartmann et al. 2014; Lupas et al. 2015). More recently, uridine, one of the pyrimidine nucleosides, was identified as the cellular ligand of cereblon, and was shown to cause teratogenicity similar to IMiDs (Hartmann et al. 2014). However, other pyrimidine nucleosides such as cytidine or thymidine are shown to have no effect on teratogenicity in vivo (Hartmann et al. 2014). Uridine is structurally similar to the glutarimide moiety of IMiDs, implying that glutarimide- or uridine- like moieties induce teratogenicity. Considering the similarity of the ligand binding sites between cereblon and other proteins containing aromatic cages, other cationic ligands such as methylated lysine and/or arginine residues and the ligands containing quaternary ammonium groups could competitively bind to the IMiDs binding pocket of cereblon.

In this study, using the crystal structures of cereblon, we carried out in silico docking simulations in order to replicate these experimental results previously reported; the enantiomeric selectivity of IMiDs, and the identification of the region of cereblon contributing to this selectivity. In addition, we aim to replicate the experimental results obtained with pyrimidine nucleosides suggesting the preference of uridine over cytidine or thymidine when binding to the cereblon.

## Methods

### In silico docking simulations

The entire docking protocol was performed using MOE 2013.08 software package (Molecular Operating Environment; Chemical Computing Group, Montreal, Quebec, Canada) (MOE 2015). 2D molecular structures of IMiDs, including (*S*)-enantiomers [(*S*)-thalidomide, (*S*)-lenalidomide and (*S*)-pomalidomide] and (*R*)-enantiomers [(*R*)-thalidomide, (*R*)-lenalidomide, (*R*)-pomalidomide], pyrimidine nucleosides (uridine, cytidine, thymidine) were obtained from the Nikkaji web service (Nikkaji 2015), and the 3D structures were modeled using the conformational search program in MOE.

The eleven crystal structures of cereblon were obtained from the Protein Data Bank (PDB) (Table 1). The Protonate3D program (Labute 2009) was used to assign ionization states, and to position hydrogen atoms into the receptor molecules. Then, after adding partial charges under the MMFF94x forcefield and fixing the backbone atoms, energy minimization of the receptors was performed. Next, the docking site of each receptor was assigned using Grid Site Finder Program (MOE 2015), which enables the selection of specific residues manually and the creation of dummy atoms docked with ligands. In this study, using this program we selected three tryptophan residues of the aromatic cage (W380, W386, W400) of the receptors.

**Table 1:** List of cereblon structures used in this study

PDB ID	Ligand	Organism
4CI1	(*S*)-thal	Chicken
4V2Y	(*S*)-thal	Bacteria
4CI2	(*S*)-len	Chicken
4TZ4	(*S*)-len	Human
4V30	(*S*)-len	Bacteria
4CI3	(*S*)-pom	Chicken
4V2Z	(*S*)-pom	Bacteria
4V31	deoxyuridine	Bacteria
4TZC	(*S*)-thal	Mouse
4TZU	(*S*)-pom	Mouse
3WX2	–	Mouse

Finally, the ligands were docked with each receptor using ASEDock program (Goto et al. 2008). During this procedure, flexibility of the ligand atoms was allowed and the backbone atoms of the receptors were tethered. Following the docking scores (U_dock), the top 10 poses of each docking pose were retained and the top scores were evaluated in terms of docking scores and poses.

## Results

### Docking results for IMiDs

**Fig. 2:**
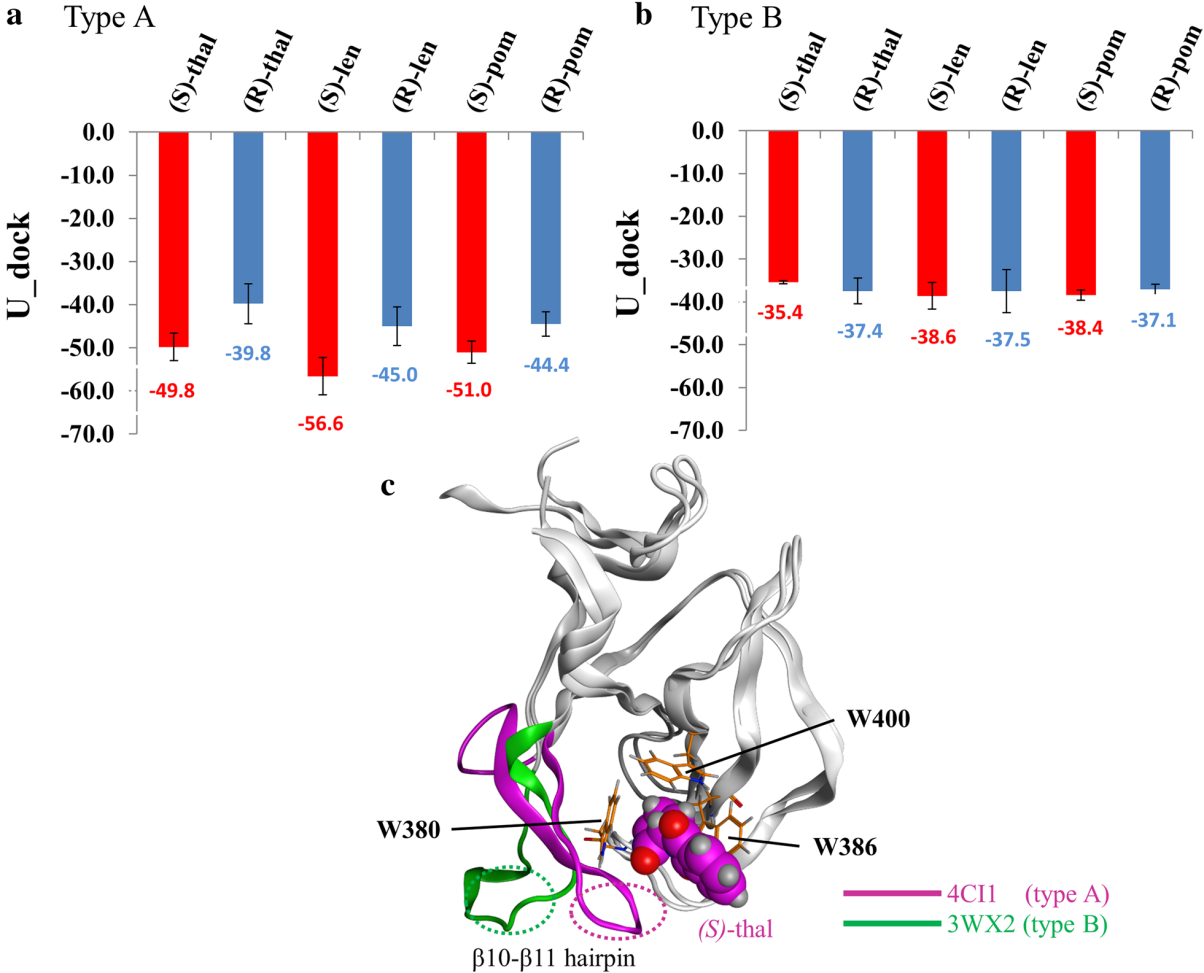
Docking results for IMiDs. Eleven cereblon structures were classified into type A (PDB ID: 4CI1, 4V2Y, 4CI2, 4TZ4, 4V30, 4CI3, 4V2Z, and 4V31) and type B (PDB ID: 4TZC, 4TZU, and 3WX2). The average docking scores for IMiDs using type A (**a**) and type B (**b**) are shown. **c** Structural differences between type A (PDB ID: 4CI1) and type B (PDB ID: 3WX2) cereblon. β10–β11 hairpin of type A and type B cereblon structures are shown in *magenta* and *green*, respectively. The three tryptophan residues of the aromatic cage (W380, W386, and W400) of cereblon and (*S*)-enantiomer of thalidomide [(*S*)-thalidomide] are shown in *orange* and *magenta*, respectively. Thalidomide, lenalidomide, and pomalidomide are described in ‘thal’, ‘len’, ‘pom’ for short, respectively

The eleven crystal structures of cereblon were obtained from the PDB (Table 1), and these structures were docked with the (*S*)-enantiomers [(*S*)-thalidomide, (*S*)-lenalidomide, and (*S*)-pomalidomide] and the (*R*)-enantiomers [(*R*)-thalidomide, (*R*)-lenalidomide, (*R*)-pomalidomide] of IMiDs. Using eight out of the eleven structures (PDB ID: 4CI1, 4V2Y, 4CI2, 4TZ4, 4V30, 4CI3, 4V2Z, 4V31), we correctly replicated the enantiomeric selectivity, where the docking scores of (*S*)-enantiomers were lower than those of the respective (*R*)-enantiomers (Fig. 2a; Additional file 1: Table S1) and the docking poses of (*S*)-enantiomers were within 2 Å of RMSD value compared to the crystal structures (Additional file 1: Figure S1). However, we could not replicate the selectivity when using the other three structures (PDB ID: 4TZC, 4TZU, 3WX2) (Fig. 2b; Additional file 1: Table S1). In these cases, some docking results were in contrast to the earlier results (PDB ID: 4TZC and 3WX2), and the overall docking scores of (*S*)-enantiomers were higher compared to the correctly replicated structures. We classified these ‘appropriate’ 8 structures and ‘inappropriate’ 3 structures into type A and type B, respectively. A detailed structural analysis revealed that compared to type A, cereblon structures of type B have some differences at the hairpin connecting β10–β11 region (residue 351–355). This hairpin region in type A is next to the IMiDs binding site, whereas this region is disordered or absent in type B structures (Fig. 2c). This is in part due to the polymerization of cereblon in crystal structures, which distorts this hairpin. This phenomenon is unlikely to occur in a natural state because no evidence of cereblon monomer polymerization has been obtained so far (Chamberlain et al. 2014). Therefore, because of this unnatural hairpin region, the docking results of type B structures might be ‘inappropriate’. Except for type B structures, we confirmed the enantiomeric selectivity of IMiDs with high accuracy. These results might be due to the increased interactions of these molecules with cereblon by their amino-phthaloyl-substituted characteristics in type A structures.

### Docking results for pyrimidine nucleosides

Next, using type A structures we also tried to replicate the selectivity of pyrimidine nucleosides i.e. the preference of uridine over cytidine and thymidine. We correctly replicated this selectivity and found that the docking scores of uridine were lower than those of the other two pyrimidine nucleosides (Additional file 1: Figure S2). As previous reports stated (Hartmann et al. 2014), these results are attributed to the structural similarity between the uracil moiety of uridine and the glutarimide moiety of IMiDs, and the structural discrimination of the pyrimidine moiety of cytidine and thymidine. The cytosine moiety of cytidine has a different functional group compared to the uracil moiety of uridine, resulting in inappropriate chemistry for the ligands to bind and in higher docking scores for cytidine. As for thymidine, the thymine moiety of the ligand has an additional methyl group compared to the uracil moiety of uridine, resulting in a steric clash with the tryptophan residues and higher docking scores for thymidine (Additional file 1: Figure S2c). Moreover, type B structures were also tested (Additional file 1: Figure S3). Because the hairpin connecting β10–β11 was incomplete or was oriented in an outer space which is further away from the TBD domain of cereblon compared with type B structures, an additional space was formed. When two of the type B structures (PDB ID: 4TZC and 4TZU) were used as receptor for docking, pyrimidine nucleosides were docked out of thalidomide binding pocket. While when type A structure (e.g. PDB ID: 4CI2) was used, nucleosides were docked into the thalidomide binding site (Additional file 1: Figure S3). One more point to mention is that according to the overlap of the structures (Additional file 1: Figure S3e), in the case of 4TZC, if a β10–β11 hairpin exists, the predicted poses should conflict with it. On the other hand, 3WX2 extended its hairpin aside compared to 4CI2, and this may due to the lack of NTD (Additional file 1: Figure S3d, e). In this case, uridine and thymidine were docked into the thalidomide binding pocket, and however cytidine was predicted in a reversed form (Additional file 1: Figure S3c). Since there was no evidence that pyrimidine nucleosides bind to a space other than thalidomide binding site, it is hard to tell the docking poses were correct and there is less meaningful to compare the docking scores of these results (Additional file 1: Table S2), but only compared the result of uridine/3WX2 with thymidine/3WX2, two poses into thalidomide binding pocket, uridine is prone to bind to cereblon, due to the score showing −40.6 versus −34.4.

## Discussion

### The β10–β11 hairpin region contributes to the enantiomeric selectivity for (*S*)-enantiomers

**Fig. 3:**
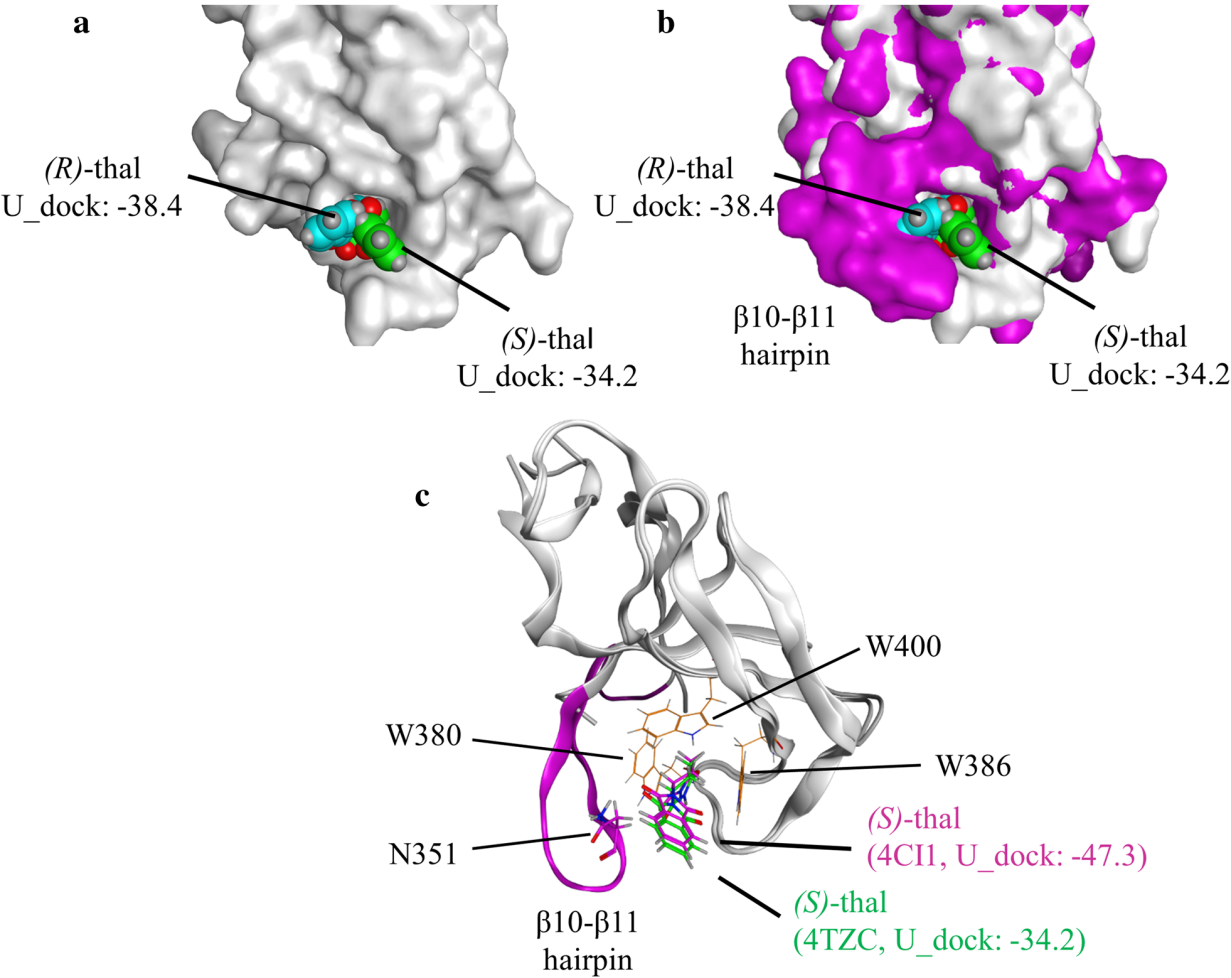
β10–β11 hairpin of cereblon could contribute to enantiomeric selectivity and the increased affinity with IMiDs. **a** Docking poses of (*S*)-thalidomide (*green*) and (*R*)-thalidomide (*cyan*) using the type B structure (*white*). **b** Superimposition of the complex shown in **a** with the type A structure (*magenta*). **c** Docking poses of (*S*)-thalidomide (*magenta*) using the type A structure and (*S*)-thalidomide (*green*) using the type B structure. β10–β11 hairpin and the three tryptophan residues of the aromatic cage (W380, W386, and W400) of type A cereblon are shown in *magenta* and *orange*, respectively. Thalidomide, lenalidomide, and pomalidomide are described in ‘thal’, ‘len’, ‘pom’ for short, respectively

 As mentioned above, the hairpin connecting β10–β11 could be a key region in the enantiomeric selectivity of IMiDs. To better understand the role of this hairpin region, we superimposed a type B cereblon structure (PDB ID: 4TZC) binding docking poses of (*S*)-thalidomide and (*R*)-thalidomide, with a type A cereblon structure (PDB ID: 4CI1) (Fig. 3a, b). These structures revealed that the (*R*)-thalidomide partially clashed with the hairpin region of 4CI1. Thus, for 4TZC the ‘inappropriate’ docking results might arise from the widely expanded binding pocket due to the disordered hairpin structure. These findings also suggest that in case of type A structures, the binding of (*R*)-enantiomers is unfavorable due to the steric hindrance caused by this hairpin, next to the IMiDs binding site. Finally, this hairpin could contribute to the IMiDs binding affinities, because the docking scores of (*S*)-enantiomers are higher for type B structures than for type A (Fig. 2; Additional file 1: Table S1). To obtain a deeper insight, we superimposed a type A cereblon structure binding the docking pose of (*S*)-thalidomide with a type B cereblon structure (PDB ID: 4TZC) binding that of (*S*)-thalidomide. These structures revealed that in absence of the β10–β11 hairpin, the interaction between the (*S*)-enantiomer and the β10–β11 hairpin constructive residues disappear (Fig. 3c). Thus, the disorder or absence of β10–β11 hairpin could result in decreasing the IMiDs binding affinity. Taken together, in addition to previously reported the aromatic cage of three tryptophan residues (W380, W386, and W400), the hairpin connecting β10–β11 may play an additional role in the recognition of IMiDs.

### The possible effects of the β10–β11 hairpin in substrate recognitions of cereblon

Cereblon, a component of DDB1/cullin4 E3 ubiquitin ligase complex, mediates the recruitment and degradation of its target proteins (Ito et al. 2011; Shortt et al. 2013). Recent experimental results imply that IMiDs act as both agonists and antagonists of cereblon (Ito et al. 2010; Krönke et al. 2014; Lu et al. 2014; Gandhi et al. 2014; Krönke et al. 2015). As an antagonist, the MEIS2 protein, which acts at various aspects of human growth (Capdevila et al. 1999), was identified as the substrate of cereblon that competitively binds to the same binding site as IMiDs (Fischer et al. 2014). As such, cereblon with bound IMiDs prevents the recruitment, ubiquitylation and degradation of MEIS2, resulting in teratogenicity (Fischer et al. 2014). As an agonist, IMiDs bound to cereblon complex promote the binding of some proteins including transcription factors implicated in multiple myeloma (such as Ikaros and Aiolos) (Krönke et al. 2014; Lu et al. 2014; Gandhi et al. 2014) or myelodysplastic syndrome (such as casein kinase 1α) (Krönke et al. 2015), which otherwise could not bind to cereblon. As a result, these events cause the degradation of these target proteins, resulting in multiple mechanisms of IMiDs action. Sequence alignment studies regarding these proteins binding to cereblon with or without IMiDs revealed that these proteins do not share high sequence homology, suggesting that the ways in which cereblon or the cereblon–IMiDs complex recognizes the substrates may have some differences. As mentioned above, the β10–β11 hairpin of cereblon has some difference between type A and type B structures due to the polymerization of cereblon in crystal structures, thus this hairpin region might play an important role with respect to recognition of substrate binding. A recent experimental study, which implies that this hairpin region only folds upon ligand binding, also supports this hypothesis (Lopez-Girona et al. 2012). We carried out a 25 ns MD computation of human cereblon structure to analyze the movement of this hairpin (Additional file 1: Figure S4). This hairpin did not move violently throughout our simulation duration as expected. It is suggested that although β10–β11 hairpin is useful in substrate recognitions, but high flexibility is not needed. Or the simulation duration was not long enough to observe the conformation change of this hairpin. Another point should be mentioned here is that all crystal structures other than the type A structures were derived from only the TBD domain of cereblon, and these structures do not cover the overall structures. More studies are required to understand the details, such as structural identification of the cereblon–MEIS2 complex, cereblon–IMiD-substrate complex, and so on.

## Conclusions

In this study, using in silico docking simulations, we aimed to confirm the enantioselective binding of IMiDs to cereblon and to identify the region of cereblon that contributes to this selectivity. For eight out of eleven crystal structures of cereblon (referred to as type A), we replicated the enantiomeric selectivity of IMiDs with the correct docking poses and scores. The results of the lower docking scores for lenalidomide and pomalidomide compared to those for thalidomide correlate well with the previous experimental studies (Fischer et al. 2014; Hartmann et al. 2015). Next, using the pyrimidine nucleosides, we performed docking simulations to replicate the previous experimental results that uridine alone binds to cereblon (Chamberlain et al. 2014; Hartmann et al. 2014). We replicated the preference of uridine to the other putative ligands with the correct docking scores.

Recent studies about the mechanism of action for cereblon have opened a new paradigm about IMiDs biology. So far, IMiDs have been considered to bind multiple targets, which lead to a variety of mechanisms, such as teratogenicity and the treatment of multiple myeloma (Ito et al. 2011; Shortt et al. 2013). In contrast, IMiDs have been recently suggested to bind to only one target, cereblon, which results in a variety of mechanisms (Handa et al. 2014). This concept of cereblon-centered mechanisms is supported by the recent findings about the cereblon-induced mechanism of action for IMiDs (Krönke et al. 2014; Lu et al. 2014; Gandhi et al. 2014; Krönke et al. 2015). Therefore, our docking results will provide novel insights into the binding mode of IMiD-like molecules and contribute to a deeper understanding of cereblon-related biology.

## Additional file


10.1186/s40064-016-2761-1 Supplementary material.

## Abbreviations

IMiDs: immunomodulatory imide drugs or IMiDs
TNF-α: tumor necrosis factor-α
NTD: the amino terminal domain
HBD: α-helical bundle domain
CBD: carboxy-terminal domain
DDB1: damage-specific DNA binding protein 1
MEIS2: myeloid ecotropoic viral insertion site homeobox 2
TBD: Tbk1/Ikki binding domain
