# Supplementary material for: In silico analysis of enantioselective binding of immunomodulatory imide drugs to cereblon
Source: Springerplus. 2016 Jul 19;5(1):1122. doi: 10.1186/s40064-016-2761-9 (PMC4949186; doi:10.1186/s40064-016-2761-9)
Supplement: Supplementary file 1 — 10.1186/s40064-016-2761-1 Supplementary material. [file 40064_2016_2761_MOESM1_ESM.pdf]

In silico analysis of enantioselective binding of immunomodulatory imide drugs to cereblon

Takahiro Murai[1], Norihito Kawashita[1,2], Yu-Shi Tian[3], Tatsuya Takagi[1,2,*]

[1] Graduate School of Pharmaceutical Sciences, Osaka University, 1-6 Yamadaoka, Suita, Osaka 565-0871, Japan

[2] Research Institute for Microbial Diseases, Osaka University, 3-1 Yamadaoka, Suita, Osaka 565-0871, Japan

[3] Graduate School of Information Science and Technology, Osaka University, 1-5 Yamadaoka, Suita, Osaka 565-0871, Japan

[*] **Corresponding author.** Graduate School of Pharmaceutical Sciences, Osaka University, 1-6 Yamadaoka, Suita, Osaka, 565-0871, Japan.

E-mail address: ttakagi@phs.osaka-u.ac.jp (T. Takagi).

Supplementary material

Supplementary material text

MD simulation

MD simulation was performed using the GROMACS 5.0.4 software package and the OPLS-AA force field. The crystal structure of human cereblon with lenalidomide was extracted from PDB (PDB ID: 4TZ4) and lenalidomide was removed from the structure to analyze the dynamics of the apo state. This structure was soaked by using the TIP3P water model. A dodecahedral box was selected with a minimum distance of 1.4 nm between the protein and the box, and the system was neutralized by adding counterions at physiological concentrations (0.15 M). The energy of the system was minimized by using the steepest descent algorithm for 100ps. The v-rescale and Parrinello–Rahman methods were utilized to control temperature and pressure, respectively. The LINCS algorithm was used to constrain all bond lengths. The particle mesh Ewald (PME) method was used to compute long-range electrostatics. Finally, 25 ns production MD simulation was performed at 310 K.

Figure Legends

Fig. S1 Superimposition of crystal structures of IMiDs with the docking poses using type A cereblon structures. Crystal structures of IMiDs and β10-β11 hairpin of the type A structure are shown in magenta, and the docking poses of IMiDs are shown in green.

Fig. S2

(a) Structure of the pyrimidine nucleosides (Uridine, Cytidine, and Thymidine). The different function group between Uridine and the other two is circled by red dot.

(b) Docking results of the pyrimidine nucleosides. The average docking scores for IMiDs and pyrimidine nucleosides using the type A cereblon are shown.

(c) Docking poses of the pyrimidine nucleosides to cereblon (PDB ID: 4CI2). The surfaces of β10-β11 hairpins are colored in magenta and the rest are colored in white. Docking poses of pyrimidines are green colored. The red circles showed the differences between Uridine and the other two pyrimidine nucleosides the same as in (a).

Fig. S3

Docking poses of pyrimidine nucleosides (Uridine, Cytidine, and Thymidine) with cereblon ((a) PDB ID: 4TZC, (b) PDB ID: 4TZU, (c) PDB ID: 3WX2, (d) PDB ID: 4CI2). Ribbon representations of cereblon were shown in green (4TZC), red (4TZU), and yellow (3WX2). For 4CI2, ribbon representation is colored according to its domain: magenta (CBD), sky blue (NTD), and bisque (HBD). Hydrogen atoms and receptor atoms were hidden, and Uridine, Cytidine, and Thymidine were colored in black, purple, and sky blue respectively. Blue dots showed a β10-β11 hairpin site. Gray circle showed an additional binding site due to the deletions of the β10-β11 hairpin. (e) A superpose of (a) to (d).

Fig. S4 25 ns MD simulation of human cereblon. The averaged rmsf values of NTD (residue 48-185), HBD (residue 186-317) and TBD (residue 318-428) of human cereblon (PDB ID: 4TZ4) are shown in green, blue and magenta, respectively. The rmsf values of β10-β11 hairpin of the protein (residue 351-355) (orange) were about 1.5 Å, suggesting that this hairpin does not show the remarkable flexibility compared to the other regions of the protein.

Fig. S1

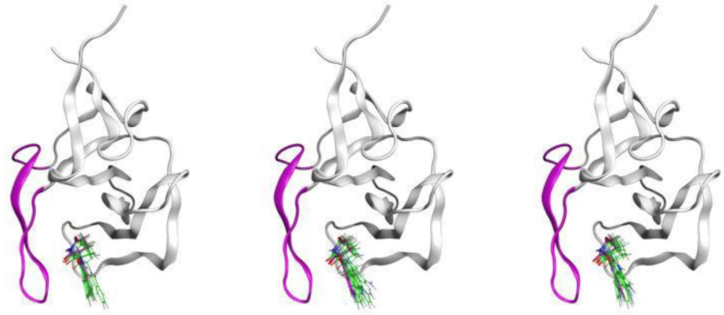

(S)-thal (S)-len (S)-pom

Fig. S2

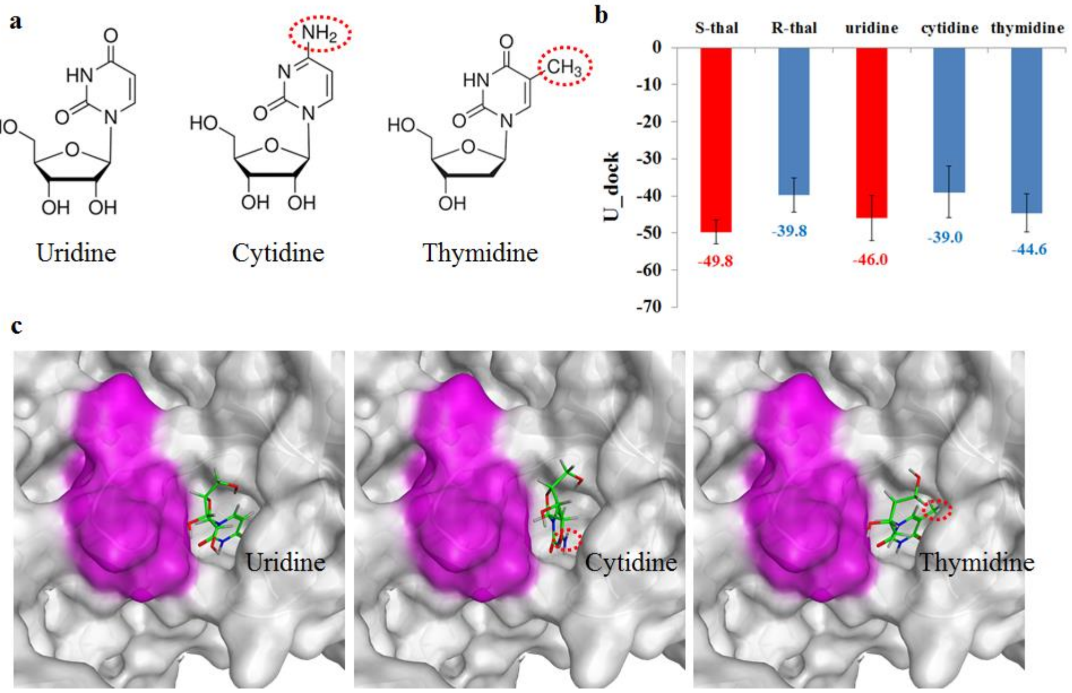
a
NH2
CH3
O
HN
N
O
HO
OH
Uridine
Cytidine
Thymidine
b
S-thal
R-thal
uridine
cytidine
thymidine
U_dock
0
-10
-20
-30
-40
-50
-60
-70
-49.8
-39.8
-46.0
-39.0
-44.6
c
Uridine
Cytidine
Thymidine

Fig. S3

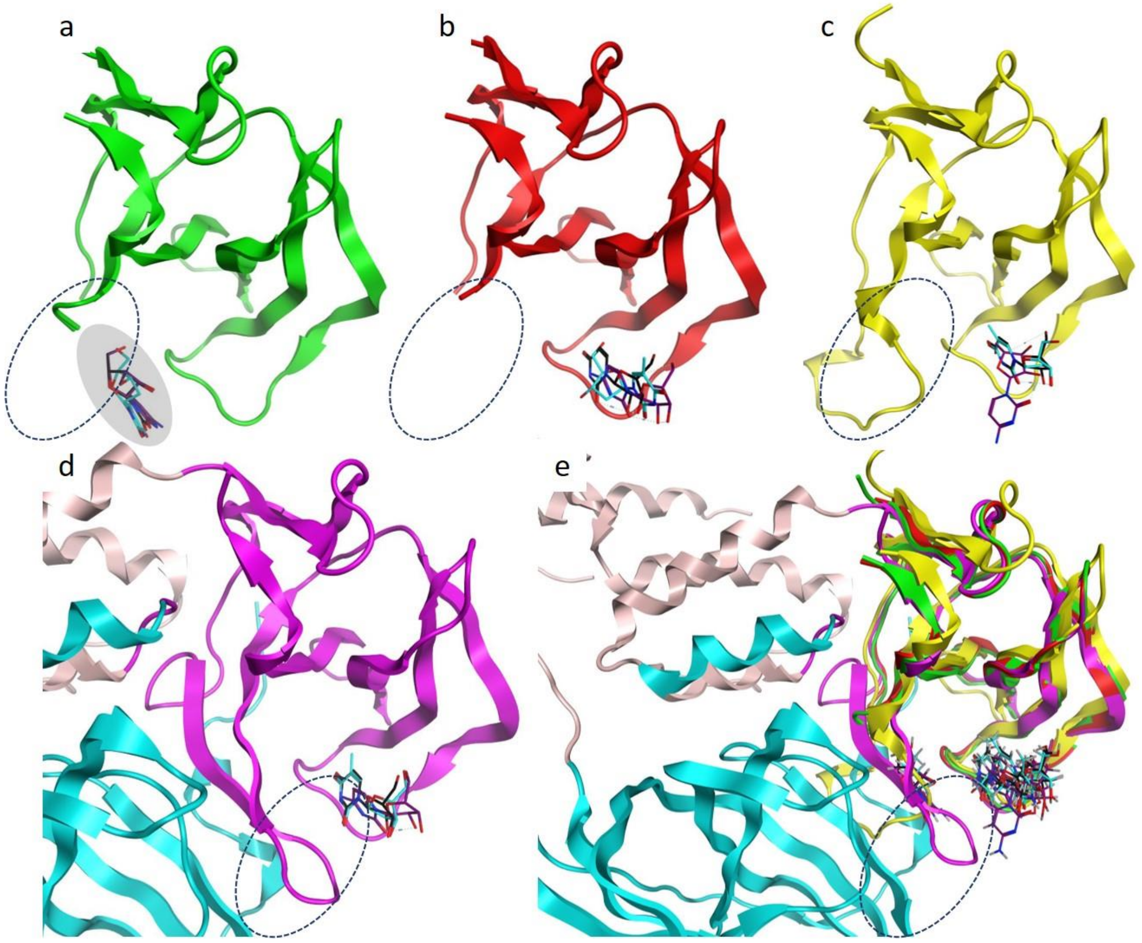

Fig. S4

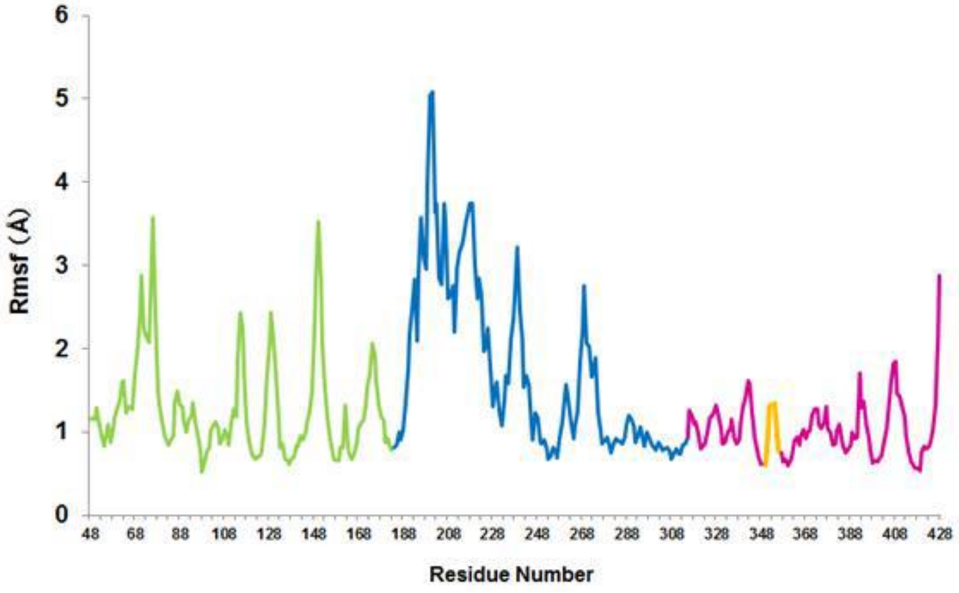
6
5
4
3
2
1
0
Rmsf (Å)
48 68 88 108 128 148 168 188 208 228 248 268 288 308 328 348 368 388 408 428
Residue Number

Tables

Table S1

Docking results of IMiDs

PDB ID	(*S*)-thal	(*R*)-thal	(*S*)-len	(*R*)-len	(*S*)-pom	(*R*)-pom	β10-β11 hairpin
4CI1	-47.3	-35.1	-52.2	-40.9	-48.2	-42.1	Type A
4V2Y	-46.6	-36.7	-57.5	-40.7	-51.8	-41.9	
4CI2	-54.0	-42.2	-60.8	-46.4	-53.2	-46.8	
4TZ4	-50.8	-43.1	-59.0	-53.1	-49.9	-45.3	
4V30	-50.6	-41.8	-54.2	-40.4	-51.8	-44.5	
4CI3	-54.6	-47.1	-63.1	-47.1	-55.6	-49.7	
4V2Z	-47.9	-32.9	-50.4	-43.2	-48.3	-43.7	
4V31	-46.6	-39.4	-55.5	-48.2	-49.5	-41.5	
4TZC	-35.5	-38.4	-38.3	-41.6	-37.6	-37.2	Type B
4TZU	-34.9	-34.0	-41.9	-31.8	-39.7	-35.8	
3WX2	-35.7	-39.9	-35.6	-39.1	-37.9	-38.3	

Table S2

Docking results of pyrimidine nucleosides into type B structures

PDB ID	Uridine	Cytidine	Thymidine	β10-β11 hairpin
**4TZC**	**-37.4**	**-39.1**	**-38.6**	**Type B**
**4TZU**	**-35.1**	**-28.6**	**-31.1**	
**3WX2**	**-40.6**	**-25.4**	**-34.4**	
